# Qualitative and Quantitative Analysis of Six Fatty Acid Amides in 11 Edible Vegetable Oils Using Liquid Chromatography–Mass Spectrometry

**DOI:** 10.3389/fnut.2022.857858

**Published:** 2022-03-28

**Authors:** Zixiang Li, Feng Dong, Yongzhi Sun, Zhaohui Sun, Xinyu Song, Yingran Dong, Xiaocai Huang, Jiayi Zhong, Rui Zhang, Maoqing Wang, Changhao Sun

**Affiliations:** National Key Disciplines of Nutrition and Food Hygiene, Department of Nutrition and Food Hygiene, School of Public Health, Harbin Medical University, Harbin, China

**Keywords:** edible vegetable oil, fatty acid amides, LC/MS, qualitative analysis, quantitative analysis, content

## Abstract

Fatty acid amides (FAAs) are endogenous lipid molecules that exhibit various physiological activities. FAAs are usually present at nanomolar levels in biological samples. In this study, a method for the qualitative and quantitative determination of six FAAs (linoleamide, linoleoyl ethanolamide, oleoyl ethanolamide, palmitic amide, oleamide, and octadecanamide) in edible vegetable oils was established. All six FAAs were detected in sesame, peanut, soybean (decolorized and non-decolorized), and blended oils; five in sunflower oil; four in rice oil; three in linseed and olive oils; and two in corn and canola oils. The total contents of FAAs were highest in sesame oil (104.88 ± 3.01 μg/mL), followed by peanut oil (34.96 ± 3.87 μg/mL), soybean oil (16.75 ± 1.27 μg/mL), and blended oil (13.33 ± 0.77 μg/mL), and the contents in the other edible vegetable oils were all <1.03 μg/mL. The concentrations of linoleoyl ethanolamide and oleoyl ethanolamide were highest in non-decolorized soybean oil, while the other four FAAs (linoleamide, palmitic amide, oleamide, and octadecanamide) showed the highest concentrations in sesame oil. The total contents of these FAAs in eight different oils were higher than those in biological fluids and tissue. Our study confirmed that edible vegetable oils are rich in FAAs, and provides reliable data for evaluating the nutritive value of vegetable oils.

## Introduction

Fatty acid amides (FAAs) are bioactive lipid signaling molecules that have a variety of physiological activities. They play key roles in biological functions, such as sleep induction, analgesic, anti-anxiety, anti-convulsion, and anti-epilepsy activities, neuroprotection, and they promote fat hydrolysis and weight loss (1–7). For example, linoleamide has been reported to exert sedative and hypnotic effects, and inhibits the migration of cancer cells in human subjects (8, 9). Linoleoyl ethanolamide can regulate pain, food intake, and blood sugar level (10, 11). Oleoyl ethanolamide can inhibit food intake, promote fat hydrolysis, and reduce body weight (12–14). Palmitic amide can induce sleep in animals and exhibit central inhibitory and anticonvulsant effects (15). Finally, oleamide has analgesic, anti-anxiety, and sleep induction activities, and can improve memory and reduce cognitive impairment, as well as preventing Alzheimer's disease, cardiovascular disease, and inflammation (5, 16–18). Owing to their broad range of bioactive functions, FAAs have generated a great deal of interest, especially in the fields of pharmacy and nutrition (19–23).

At present, FAAs are generally considered to be endogenous lipid molecules that are found mainly in mammals, such as cats, squirrels, and humans (19, 24–27). However, their concentrations in tissues and biological fluids of mammals are low, typically in the nanomolar range (26, 28). As a result, the extraction and purification of FAAs from tissues or organs is challenging.

Palmitic amide has been found in soybeans, peanut oil, and egg whites (29) and palmitic amide, oleamide, and N-acylethanolamines have been detected in different edible vegetable oils (30–33). In our previous study, we used liquid chromatography–mass spectrometry (LC–MS) to identify six FAAs in peanut oil: linoleoyl ethanolamide, linoleamide, oleoyl ethanolamide, palmitic amide, oleamide, and octadecanamide (34). This work showed that edible vegetable oils may be rich in FAAs. If enough FAAs are isolated or consumed from vegetable oils, they may play important roles in clinical treatment or nutritional intervention. Therefore, it is important to identify the types of FAAs present in different edible vegetable oils and to determine their contents. However, no qualitative and quantitative method of analysis for FAAs is available.

Therefore, this study aimed to establish a qualitative and quantitative method for the simultaneous determination of the six FAAs using ultra-performance liquid chromatography–tandem quadrupole time-of-flight mass spectrometry (UPLC/Q-TOF MSMS). The proposed method was used to identify FAAs and compare their concentrations in 11 edible vegetable oils, thus providing a basis for evaluating the nutritional value of different edible vegetable oils.

## Materials and Methods

### Materials and Reagents

Decolorized and non-decolorized soybean oils (Jiusan Oils and Grains Industries Group Co., Ltd., Heilongjiang, China), sesame oil (Liaoning Qingyan Temple Food Co., Ltd., Liaoning, China), peanut oil (Shandong Luhua Group Co., Ltd., Shandong, China), canola oil (Hulun Buir Heshijia Food Co., Ltd., Inner Mongolia, China), corn oil (Xiwang Foodstuffs Co., Ltd., Shandong, China), linseed oil (Ngol League Hong Jing Yuan Grease Co., Ltd., Inner Mongolia, China), olive oil (Mueloliva, Cordoba, Spain), rice oil (China Resources Ng Fung, Shenzhen, China), sunflower oil (Shanghai Standard Foods Co., Ltd., Shanghai, China), and blended oil (sunflower oil 25%, canola oil 24%, corn oil 18.9%, soybean oil 18%, peanut oil 6%, rice oil 5%, linseed oil 2.5%, sesame oil 0.6%; Yihai Kerry Arawana Holdings Co., Ltd, Shanghai, China) were purchased in local supermarkets in Harbin, China.

Acetonitrile and methanol (Fisher Scientific, Suwannee, GA, USA), and formic acid (Tianjin Institute of Fine Chemicals, Tianjin, China) were of chromatography grade. Ultra-pure water was obtained from a water purification system (Milli-Q, Milford, MA, USA). Standard compounds, linoleoyl ethanolamide, linoleamide, oleoyl ethanolamide, palmitic amide, oleamide, and octadecanamide (purity ≥98%) were obtained from Shanghai ZZBio (Shanghai, China). Stock solutions of the six FAAs (100 μg/mL) were prepared with methanol and stored in amber glass vials at −20°C until analysis.

### Experimental Methods

#### Sample Pretreatment

A sample of vegetable oil (0.8 mL) was put into a 15-mL Eppendorf tube with 1.6 mL of methanol solution, mixed by vortexing for 3 min (Scientific Industries Inc., Bohemia, NY, USA), and then centrifuged at 58.3 ×g for 10 min in a refrigerated high-speed centrifuge (Eppendorf AG, Hamburg, Germany). The supernatant (400 μL) was removed, added to a sample bottle, and then analyzed by ultra-performance liquid chromatography (ACQUITY UPLC System, Waters, Milford, MA, USA) coupled with mass spectrometric detection (Micromass Q-Tof Micro; Waters, Wilmslow, UK).

#### UPLC Conditions

Analysis of the composition of peanut oil in our previous study showed that the six FAAs could be well separated using an ACQUITY UPLC BEH C18 column (100 × 2.1 mm; 1.7 μm, Waters) (34). Therefore, the same column and chromatographic conditions were used to separate the six FAAs in the 11 edible vegetable oils investigated in the present study. For the UPLC chromatographic separation, the column temperature was 35°C, the sample chamber temperature was 4°C, the mobile phase flow rate was 350 μL/min, and the injection volume was 4 μL. Mobile phase A was ultrapure water containing 0.1% formic acid and mobile phase B was acetonitrile. A linear gradient elution procedure was performed as follows: 0–0.5 min, 98% A; 0.5–3 min, 98–30% A; 3–10.5 min, 30–2% A; 10.5–12 min, 2% A; 12–14 min, 2–98% A; 14–16 min, 98% A.

#### Mass Spectrometry Conditions

The mass spectrometer (Micromass Q-Tof Micro, Waters) interfaced with the ESI source was operated in the positive ion mode (ESI+). The system analytical parameters were as follows: capillary voltage, 3.0 kV; cone hole voltage, 35 V; source temperature, 125°C; cone gas flow (nitrogen), 50 L/h; desolvation temperature, 320°C; desolvation gas (nitrogen) flow, 720 L/h; collision gas, argon; microchannel plate detector voltage, 2400 V. Leucine-enkephalin (Waters) was used as the lock mass (m/z 556.2771) in ESI+ at a concentration of 200 pg/mL. The data were collected in centroid mode using the full scan mode at a mass ratio of m/z 50–1,000, and the data were collected from 0 to 16 min. The Q-TOF mass acquisition rate was set at 0.4 with an interscan delay of 0.1 s.

#### Qualitative and Quantitative Method

The retention times and the parent ion peaks of the different FAAs were obtained by first-order mass spectrometry of the six FAA standard solutions. The optimal secondary mass spectra (MSMS) of the different FAAs were obtained by optimizing the collision energy. The characteristic fragment ions of the six FAAs were selected as the qualitative ions. The types of potential FAAs in different edible vegetable oils were qualitatively analyzed by comparing their retention times, the mass charge ratios (m/z) of the parentions, and the characteristic fragment ions of the standard substances.

Standard solutions of the six FAAs were prepared by diluting the standard solutions with methanol. The linear measuring ranges were evaluated by constructing standard curves for each standard.

### Evaluationof Methodology

#### Recovery Rate of Method

The recoveries of the added standards were assessed by comparing the concentrations of the six FAAs in peanut oil before and after the addition of specific amounts of FAAs. The mixed standard solutions of the six FAAs at low, medium, and high concentrations (0.1, 0.5, and 1 μg/mL) were added to the peanut oil. Six parallel samples were made for each concentration. The samples were prepared as described in Sample Pretreatment, and the concentrations were calculated by quantitative analysis as described below. Peanut oil with no added standard solution was also analyzed six times in parallel, and the concentrations of the six FAAs were calculated. The recoveries of the added standards were calculated according to the formula:


$$\begin{array}{r} \begin{array}{c} Recovery\;of\;added\;standard\;\left(\%\right)=\\ \;\left(C_{added\;sample}-C_{unadded\;sample}\right)/C_{added\;sample\;}\times \;100\% \end{array} \end{array}$$

where C_added sample_ is the concentration of sample with the added standards; C_unadded sample_ is the concentration of the sample without the added standards.

#### Precision of Method

Replicate analyses (*n* = 6) of three QC samples were conducted to assess the intra-assay and inter-day precision and accuracy. The precision was evaluated by the coefficient of variation (CV%) and the accuracy was calculated as the bias or percentage deviation between the nominal and measured concentrations.

#### Limits of Detection and Quantitation

The limits of detection (LOD) and quantitation (LOQ) of the six different FAAs were calculated as the concentration that provided signals three and ten times higher than the background noise, respectively, measured at a time close to each chromatographic signal.

### Qualitative and Quantitative Analysis of FAAs in Edible Vegetable Oils

Using the established method, six FAAs in 11 common edible vegetable oils were qualitatively and quantitatively analyzed. Each sample was measured three times in parallel. The contents of the different FAAs in each sample were calculated from the standard working curve.

## Results

### Chromatography and Mass Spectrometry

**Figure 1:**
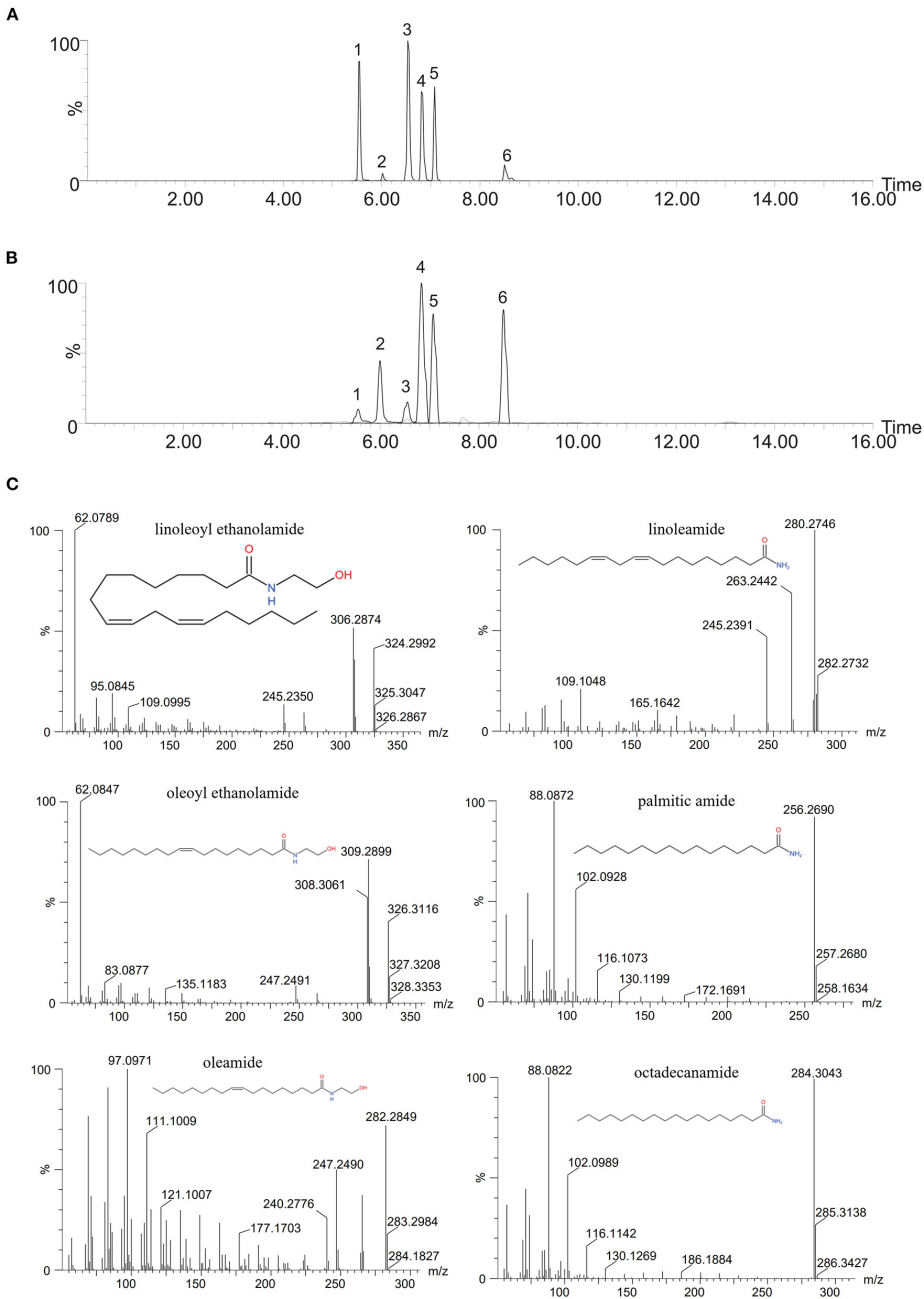
Base peak intensity ion chromatograms and MSMS spectra of six FAAs in standard solution and vegetable oil. **(A)** Six FAAs in standard solutions; **(B)** six FAAs in sesame oil. 1: Linoleoyl ethanolamide, 2: linoleamide, 3: oleoyl ethanolamide, 4: palmitic amide, 5: oleamide, 6: octadecanamide. **(C)** MSMS spectra.

As shown in Figure 1, the six types of FAA were well separated under the chromatographic conditions, confirming that the method can be used to separate and detect FAAs in vegetable oil.

The optimal secondary mass spectra of each FAA were obtained by optimizing the collision energy, with the two characteristic ions with the highest abundance selected as the qualitative ions. The parent ions, qualitative ions, and mass spectrum parameters of the six FAAs are shown in Table 1. These data can be used for qualitative analysis of the target compounds in different edible vegetable oils.

**Table 1:** The parent ions, qualitative ions and mass spectrum parameters of six fatty acid amides.

**Compounds**	**Retention time (min)**	**Formula**	**Experimental** **(m/z)**	**Theoretical** **(m/z)**	**Error** **(ppm)**	**Fragment ions** **(m/z)**	**Collision energy** **(eV)**
Linoleoyl ethanolamide	5.55	C_20_H_37_NO_2_	324.2878	324.2903	−7.71	306.2874, 109.0990	21
Linoleamide	6.01	C_18_H_33_NO	280.2665	280.2640	8.92	263.2442, 95.0861	22
Oleoyl ethanolamide	6.57	C_20_H_39_NO_2_	326.3076	326.3059	5.21	309.2899, 247.2491	24
Palmitic amide	6.86	C_16_H_33_NO	256.2629	256.2640	−4.29	130.1205, 88.0879	22
Oleamide	7.10	C_18_H_35_NO	282.2771	282.2797	−9.21	265.2612, 111.1009	24
Octadecanamide	8.51	C_18_H_37_NO	284.2966	284.2953	4.57	102.0989, 71.0840	25

### Method Evaluation

#### Standard Curves, Limits of Detection, and Limits of Quantitation

Table 2 shows that the linear relationships of the six FAAs were good over the stated linear ranges, with all R2 values >0.99. The LODs were 0.001–0.01 μg/mL and the LOQs were 0.005–0.02 μg/mL.

**Table 2:** Linear regression equations, linear ranges, detection limits and quantitation limits of standard curves for six fatty acid amides.

**Compounds**	**Linear regression equation**	**R^**2**^**	**Linear range** **(μg/mL)**	**LOD** **(μg/mL)**	**LOQ** **(μg/mL)**
Linoleoyl ethanolamide	Y = 227.48x + 9.2634	0.9971	0.05-5	0.01	0.02
Linoleamide	Y = 9.6599x – 1.6774	0.9942	0.08-8	0.008	0.05
Oleoyl ethanolamide	Y = 668.89x – 3.3845	0.9987	0.03-2	0.001	0.008
Palmitic amide	Y = 923.11x + 189.03	0.9925	0.04-2	0.001	0.005
Oleamide	Y = 111.87x + 133.84	0.9924	0.05-10	0.002	0.008
Octadecanamide	Y = 778.18x + 113.73	0.9916	0.04-1	0.002	0.008

#### Recovery of Added Standards and Method Precision

Supplementary Table 1 shows that the recoveries of the added standards of the six FAAs were in the range of 86.2–96.0%, and the relative standard deviations (RSD) from six measurements were in the range of 3.6–11.3%. Supplementary Table 2 shows that the RSD for intra-day precision ranged from 3.5 to 7.7%, and the RSD of inter-day precision ranged from 6.2 to 11.3%. These results indicated that the established method has good accuracy and precision, and can be used for the quantitative analysis of FAAs in edible vegetable oils.

#### Qualitative and Quantitative Analysis of FAAs in Edible Vegetable Oils

Using the established method, qualitative and quantitative analyses of the six FAAs in 11 edible vegetable oils were conducted. Table 3 shows that all six FAAs were detected in sesame, peanut, soybean (decolorized and non-decolorized), and blended oils; five were detected in the rice and sunflower oils, three in the olive and linseed oils, and two in the corn and canola oils.

**Table 3:** The content of six fatty acid amides found in 11 types of edible vegetable oil (μg/mL).

**Oils**	**Linoleoyl ethanolamide**	**Linoleamide**	**Oleoyl ethanolamide**	**Palmitic amide**	**Oleamide**	**Octadecanamide**	**Total amide concentration**
Sesame oil	1.62 ± 0.16	20.41 ± 1.10	1.49 ± 0.24	6.76 ± 0.48	43.92 ± 0.78	10.69 ± 0.01	84.89
Peanut oil	2.50 ± 0.25	8.50 ± 0.96	1.51 ± 0.15	3.06 ± 0.40	17.49 ± 2.14	1.90 ± 0.03	34.96
Soybean oil (non-decolorized)	7.28 ± 0.70	5.50 ± 0.26	1.52 ± 0.12	0.93 ± 0.23	1.22 ± 0.02	0.31 ± 0.03	16.76
Blended oil	1.06 ± 0.14	5.56 ± 0.94	0.53 ± 0.04	1.10 ± 0.06	4.38 ± 0.25	0.72 ± 0.06	13.35
Soybean oil (decolorized)	2.40 ± 0.07	1.84 ± 0.47	0.64 ± 0.08	0.10 ± 0.001	0.41 ± 0.06	0.07 ± 0.001	5.46
Sunflower oil	0.10 ± 0.01	–	0.06 ± 0.001	0.05 ± 0.011	0.21 ± 0.03	0.07 ± 0.004	0.49
Rice oil	0.14 ± 0.004	–	0.11 ± 0.01	0.02 ± 0.003	–	0.03 ± 0.002	0.3
Linseed oil	–	–	–	0.14 ± 0.03	0.52 ± 0.04	0.37 ± 0.02	1.03
Olive oil	–	–	0.33 ± 0.03	0.02 ± 0.004	–	0.04 ± 0.001	0.39
Corn oil	0.14 ± 0.01	–	0.09 ± 0.001	–	–	–	0.23
Canola oil	–	–	0.06 ± 0.001	–	–	0.04 ± 0.001	0.1

*“-”Indicates no FAAs were detected or below the detection limit*.

The quantitative results indicated that the contents of FAAs varied among the different vegetable oils. Of the 11 edible vegetable oils, the total concentration of FAAs was the highest in sesame oil (84.89 μg/mL), followed by peanut oil (34.96 μg/mL) and soybean oil (non-decolorized) (16.76 μg/mL). The total concentrations of FAAs in the sunflower, rice, linseed, olive, corn, and canola oils were all <1.03 μg/mL, with that in canola oil being the lowest.

The concentrations of linoleamide, palmitic amide, oleamide, and octadecanamide were highest in sesame oil, and were all more than twice those in the other edible vegetable oils. The concentrations of linoleoyl ethanolamide and oleoyl ethanolamide were highest in soybean oil (non-decolorized).

## Discussion

Because of its advantages of rapid analysis, high sensitivity, and qualitative and quantitative accuracy, LC–MS has been used in a variety of fields to determine the contents of various nutrients in edible vegetable oils (35–37) and in heated vegetable oils used for frying (38, 39). In the present study, we have established for the first time a qualitative and quantitative method for determining six types of FAA in edible vegetable oils. The retention time and characteristic ions were used to identify the amides in the qualitative results by eliminating interference caused by chromatographic time drift. Therefore, our established method will be of great use for the accurate quantitative analysis of different FAAs in edible vegetable oils.

**Figure 2:**
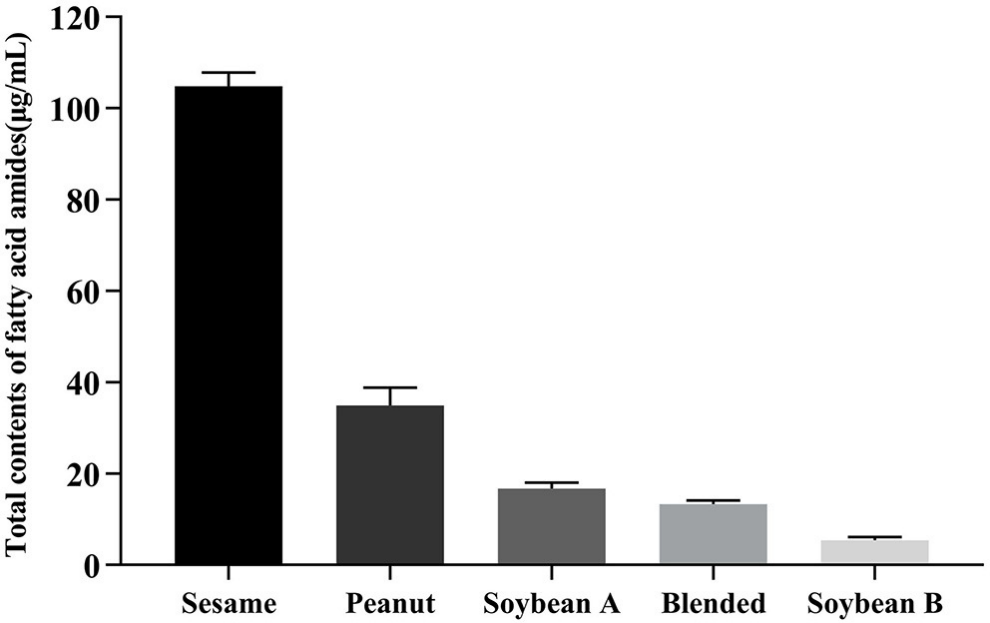
Total contents of six FFA in five edible vegetable oils. Soybean A: non-decolorized; soybean B: decolorized.

Our quantitative results showed that of the six FAAs analyzed in this study, two were detected in all 11 edible vegetable oils. The total contents of FAAs in eight different oils were higher than their concentrations in tissue and biological fluids (>1 nM). Therefore, our results indicated that edible vegetable oils, especially sesame, soybean, and peanut oils, are rich in FAAs, although the concentrations of the six FAAs varied in different vegetable oils. Sesame oil, peanut oil, the two types of soybean oil, and blended oil not only contained all six types of FAA, but also contained high total concentrations (Figure 2). Notably, the concentration of FAAs in decolorized soybean oil (5.46 μg/mL) was significantly lower than that in non-decolorized soybean oil (16.76 μg/mL), but the proportions of the six types of FAA were similarin both soybean oils. Nutrients, such as squalene, tocopherol, sterol, and carotenoid, have been reported to be lost or to decrease during the decolorization and deodorization processes used for edible vegetable oil (40, 41). Our results suggest that the refining process for edible oil reduced the contents of FAAs in decolorized soybean oil. Therefore, the refining process must be improved to retain nutrients to the greatest extent while still removing any harmful substances.

The blended oil used in the present study included soybean, peanut, sesame, and other edible vegetable oils in given proportions, and thus the six FAAs were also detected in the blended oil. Our results demonstrated that mixing different vegetable oils improves their nutritional value. The present study has also provided valuable information on the concentrations of FAAs in different vegetable oils that can aid in the preparation of blended oils.

The present study found that sesame oil contained six types of FAA, with higher concentrations of linoleamide, palmitic amide, oleamide, and octadecanamide than in the other vegetable oils. There sults suggested that these high concentrations of FAAs were responsible for the high nutritional value and good physiological functions of sesame oil. However, sesame oil is used mainly as a seasoning oil for culinary purposes, and therefore, total dietary intake tends to be relatively low. This means that its contribution in terms of dietary intake of FAAs is low. In contrast, peanut and soybean oils are used much more often and in larger amounts in daily life, and therefore their contributions in terms of dietary intake of FAAs are relatively high. Therefore, to ensure adequate dietary intake of FAAs, it is recommended that the availability of FAA-rich oils be promoted by increasing production of peanut and non-decolorized soybean oil, as well as blended oils rich in sesame and peanut oils.

## Conclusion

Our established analytical method can be used for qualitative and quantitative analysis of the FAAs in a wide range of edible vegetable oils. Our study confirmed that edible vegetable oils, especially sesame, soybean, and peanut oils, are rich in FAAs. This work provides a reference for the detection of FAAs in edible vegetable oils and provides a scientific basis for further evaluation of the nutritional value, production technology, improved blending, and consumption patterns of edible vegetable oils.

## Data Availability Statement

The original contributions presented in the study are included in the article/Supplementary Material, further inquiries can be directed to the corresponding authors.

## Author Contributions

MW and CS contributed to the study's conception and design. ZL and FD drafted and revised the manuscript and performed experimental work. FD, YS, ZS, and XS performed data collection and data analysis. YD, XH, JZ, and RZ performed data verification and data presentation. The final draft was read and approved by all authors.

## Funding

This work was supported by a grant from National Nature Science Foundation of China [81973036].

## Conflict of Interest

The authors declare that the research was conducted in the absence of any commercial or financial relationships that could be construed as a potential conflict of interest.

## Publisher's Note

All claims expressed in this article are solely those of the authors and do not necessarily represent those of their affiliated organizations, or those of the publisher, the editors and the reviewers. Any product that may be evaluated in this article, or claim that may be made by its manufacturer, is not guaranteed or endorsed by the publisher.
