# Supplementary material for: Qualitative and Quantitative Analysis of Six Fatty Acid Amides in 11 Edible Vegetable Oils Using Liquid Chromatography–Mass Spectrometry
Source: Front Nutr. 2022 Mar 28;9:857858. doi: 10.3389/fnut.2022.857858 (PMC8997291; doi:10.3389/fnut.2022.857858)
Supplement: Supplementary file 1 [file Table_1.docx]

**Supplementary material 1 Recoveries of added standards of six fatty acid amides (n=6, % )**

| **Compounds** | **0.1 µg/mL** | | **0.5 µg/mL** | | **1 µg/mL** | |
| --- | --- | --- | --- | --- | --- | --- |
|  | **Recovery** | **RSD** | **Recovery** | **RSD** | **Recovery** | **RSD** |
| **Linoleoyl ethanolamide** | 89.7 | 5.8 | 91.3 | 4.2 | 90.8 | 3.6 |
| **Linoleamide** | 91.8 | 5.3 | 93.6 | 6.1 | 86.2 | 4.6 |
| **Oleoyl ethanolamide** | 93.3 | 9.8 | 93.7 | 5.4 | 88.7 | 4.7 |
| **Palmitic amide** | 88.9 | 8.2 | 90.0 | 10.4 | 89.2 | 7.8 |
| **Oleamide** | 90.4 | 9.5 | 90.2 | 10.1 | 96.0 | 11.3 |
| **Octadecanamide** | 95.3 | 4.8 | 94.5 | 5.7 | 90.6 | 3.9 |

**Supplementary material 2 Intra-day and inter-day precision of six fatty acid amides (n=6, % )**

| **Compounds** | **0.1 µg/mL** | | **0.5 µg/mL** | | **1 µg/mL** | |
| --- | --- | --- | --- | --- | --- | --- |
|  | **intra** | **inter** | **intra** | **inter** | **intra** | **inter** |
| **Linoleoyl ethanolamide** | 6.9 | 9.4 | 6.5 | 8.8 | 3.5 | 6.2 |
| **Linoleamide** | 7.7 | 10.3 | 4.4 | 7.9 | 5.2 | 8.6 |
| **Oleoyl ethanolamide** | 4.6 | 10.1 | 7.2 | 11.3 | 3.6 | 7.3 |
| **Palmitic amide** | 4.5 | 9.7 | 4.8 | 8.5 | 5.7 | 8.9 |
| **Oleamide** | 4.8 | 7.6 | 5.2 | 7.9 | 6.6 | 10.1 |
| **Octadecanamide** | 7.5 | 10.8 | 5.3 | 9.4 | 4.5 | 8.2 |
